# Supplementary material for: Input-driven chaotic dynamics in vortex spin-torque oscillator
Source: Sci Rep. 2022 Dec 15;12:21651. doi: 10.1038/s41598-022-26018-z (PMC9755258; doi:10.1038/s41598-022-26018-z)
Supplement: Supplementary file 1 — Supplementary Information. [file 41598_2022_26018_MOESM1_ESM.pdf]

Input-driven chaotic dynamics in vortex spin-torque oscillator

Yusuke Imai[1], Kohei Nakajima[2], Sumito Tsunegi[1,3], and Tomohiro Taniguchi[1,*]

[1]National Institute of Advanced Industrial Science and Technology (AIST), Research Center for Emerging Computing Technologies, Tsukuba, Ibaraki 305-8568, Japan
[2]Graduate School of Information Science and Technology, The University of Tokyo, Bunkyo-ku, 113-8656 Tokyo, Japan
[3]PRESTO, Japan Science and Technology Agency (JST), Saitama 332-0012, Japan
[*]tomohiro-taniguchi@aist.go.jp

ABSTRACT

In this Supplementary Information, we provide the dependencies of the Lyapunov exponent and the short-term memory capacity on system parameters, h_x and ζ, for various current I and pulse width t_p. We also show a correspondence between the Lyapunov exponents and a bifurcation diagram of the vortex dynamics. The short-term memory capacity and the correlation coefficients near the edge of chaos are also shown.

Lyapunov exponent and short-term memory capacity on various parameters

In Fig. 3(a) in the main text, we show the dependence of the Lyapunov exponent on the input strength h_x and the amplitude-frequency coupling parameter ζ for $0 \le h_x \le 16$ Oe and $0 \le \zeta \le 8.0$. Our argument around that is that the Lyapunov exponent becomes negatively large when h_x and/or ζ increasing for small h_x and ζ. The validity of the argument might be, however, unclear from the figure. Therefore, in Fig. S1(a) here, we show an enlarged view of the Lyapunov exponent, where $0 \le h_x \le 2$ Oe. The current and the pulse width is 2.5 mA and 3.0 ns, respectively. The negative enhancement of the Lyapunov exponent with h_x increasing can be seen, for example around $\zeta \simeq 1.0$ and $0 \le h_x \le 0.3$ Oe. Another argument in the main text is that the short-term memory capacity is finite (zero) when the Lyapunov exponent is negative (positive). In the main text, since the Lyapunov exponent is mainly positive in Fig. 3(a), the short-term memory capacity is also mainly zero, as shown in Fig. 4(d). In Fig. S1(b) shown here, on the other hand, a finite short-term memory capacity is found in a small h_x region.

In the main text, the pulse width t_p is fixed to 3.0 ns. Here, we show that the arguments made in the main text are valid for different values of t_p. Figures S2(a)-S2(c) summarize the dependencies of the Lyapunov exponent on h_x and ζ for $t_p = 10.0$ ns and the current I is (a) 2.5, (b) 4.0, and (c) 5.0 mA, respectively. The short-term memory capacity in these parameters are also shown in Figs. S2(d)-S2(f). Similarly, those for the pulse width of 30.0 ns are summarized in Fig. S3. The correspondence between the Lyapunov exponent and the short-term memory capacity described in the main text is also verified in these cases, i.e., the short-term memory capacity is finite (zero) when the Lyapunov exponent is negative (positive). We also notice that the maximum value of the short-term memory capacity could be large compared to that found for the pulse width of 3.0 ns; see also Figs. 4(d)-4(f) in the main text. This is because the amount of information on the input data stored in the dynamical response could be large when the pulse width become long[1].

Lyapunov exponent and bifurcation diagram

The dependence of the Lyapunov exponent on the system parameters, such as h_x and ζ, sometimes show non-monotonic behavior. For example, as written in the main text, the Lyapunov exponent as a function of ζ in Fig. 3(c) is either be positive or negative when ζ is around 3.0. Similar behaviors are observed in, for examples, Figs. S2(b), S2(c), S3(b), and S3(c) here. Such a change of the sign in the Lyapunov exponent indicates that the dynamical state of the vortex core shows transitions between ordered and chaotic dynamics. To verify such a transition from a different viewpoint, we evaluate the bifurcation diagram of the distance $|\mathbf{X}_1 - \mathbf{X}_2|/R$ between two solutions of the Thiele equation with slightly different initial conditions. We calculate the temporal dynamics of $\mathbf{X}_1$ and $\mathbf{X}_2$ and define d as local maxima of the distance. Figure S4 summarize d as a function of ζ, where $I = 5.0$ mA, $h_x = 16.0$ Oe, and $t_p = 30.0$ ns. Note that d is zero when the input-driven synchronization occurs, whereas $d \neq 0$ when the synchronization is not realized. The Lyapunov exponent is also shown in the figure. A correspondence between d and the Lyapunov exponent can be seen, where d is zero (finite) when the Lyapunov exponent is negative (positive). A structure appeared in the bifurcation diagram is a window, where an ordered state appears in parameter

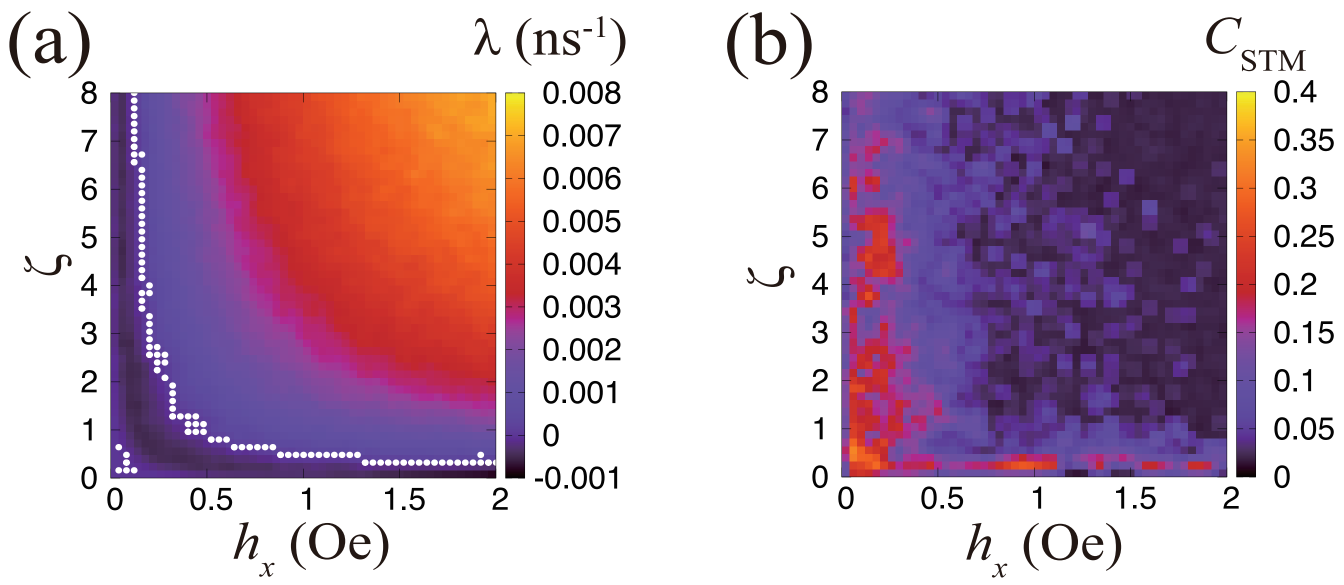

Figure S 1. Enlarged views of (a) Lyapunov exponent (λ) and (b) short-term memory capacity (C_{STM}) as functions of the input strength h_x and the amplitude-frequency coupling parameter ζ; see Figs. 3(a) and 4(d) in the main text for comparison, where the range of h_x is wider than that here. The white dots represent the zero-exponent regions.

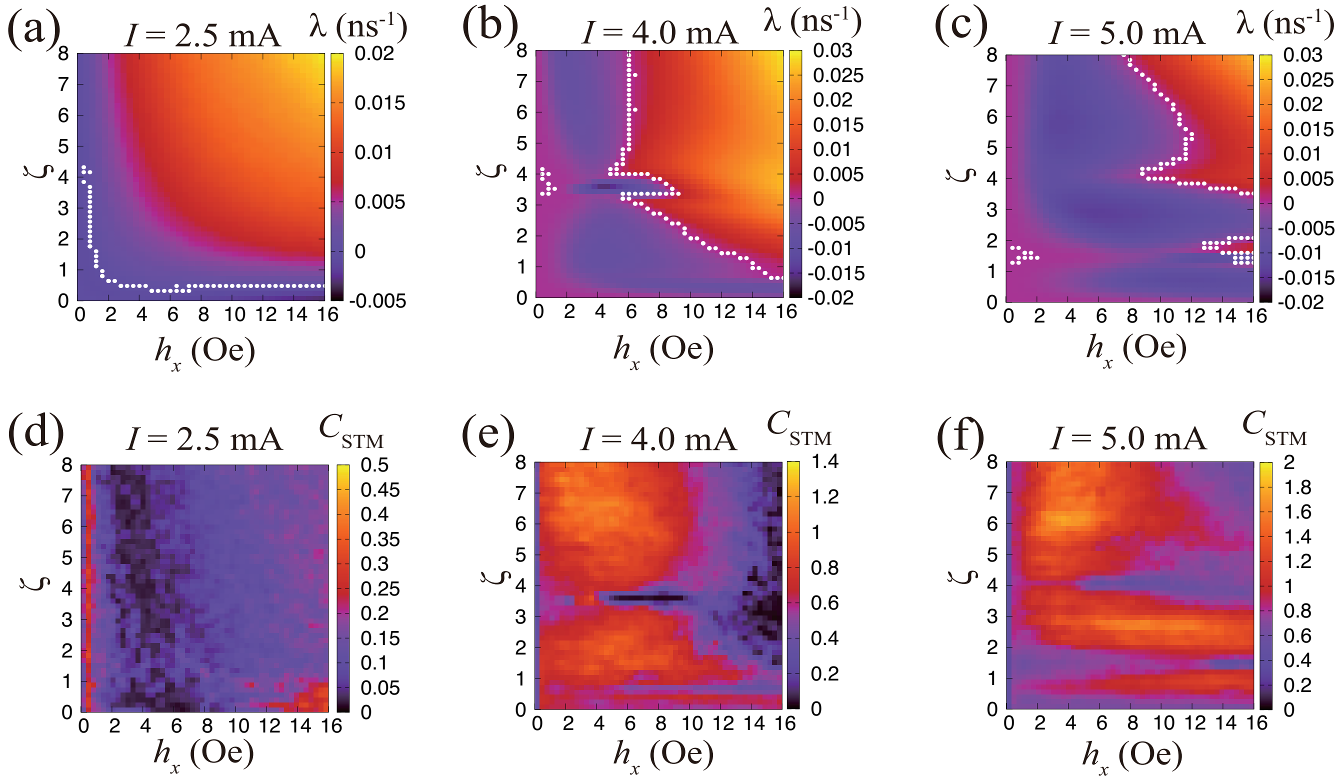

Figure S 2. (a)-(c) Lyapunov exponent and (d)-(f) short-term memory capacity for the pulse width of 10.0 ns. The values of current are (a),(d) 2.5, (b),(e) 4.0, and (c),(f) 5.0 mA. The white dots represent the zero-exponent regions.

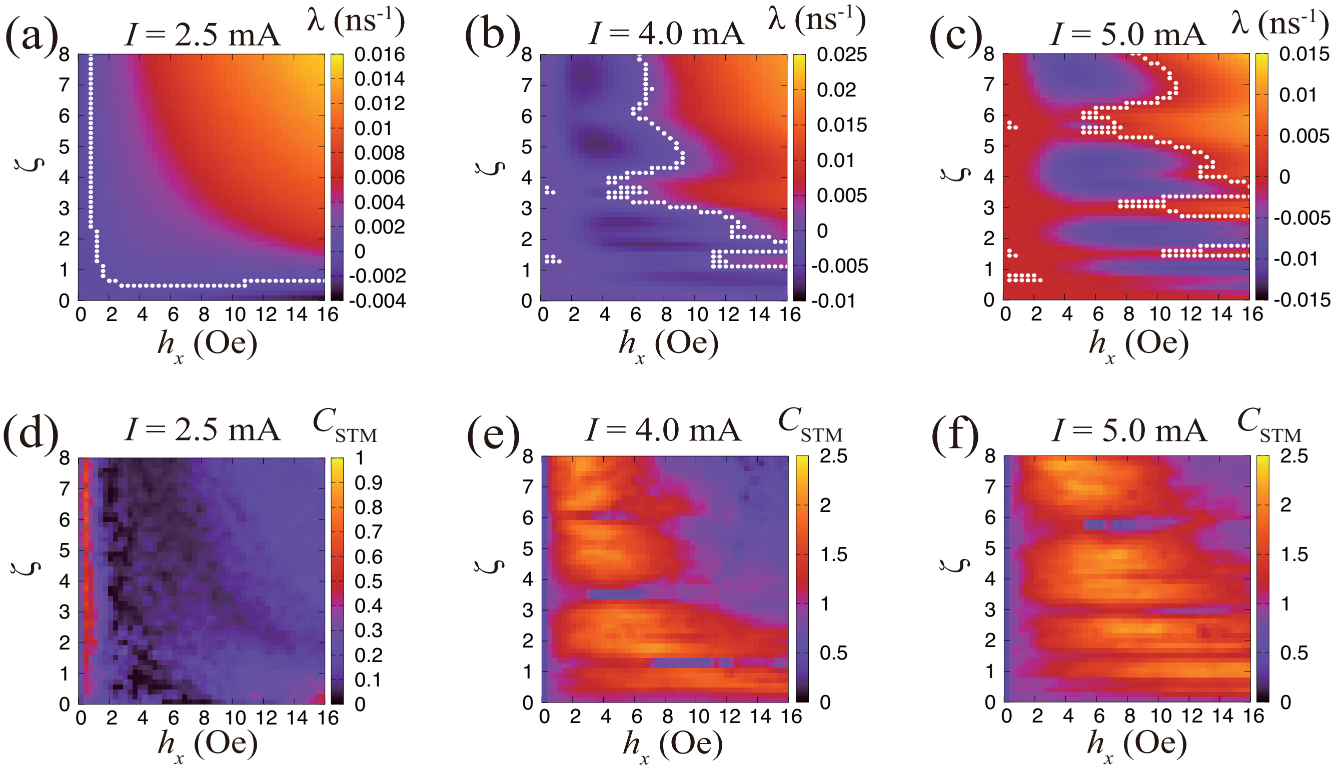

Figure S 3. (a)-(c) Lyapunov exponent and (d)-(f) short-term memory capacity for the pulse width of 30.0 ns. The values of current are (a),(d) 2.5, (b),(e) 4.0, and (c),(f) 5.0 mA. The white dots represent the zero-exponent regions.

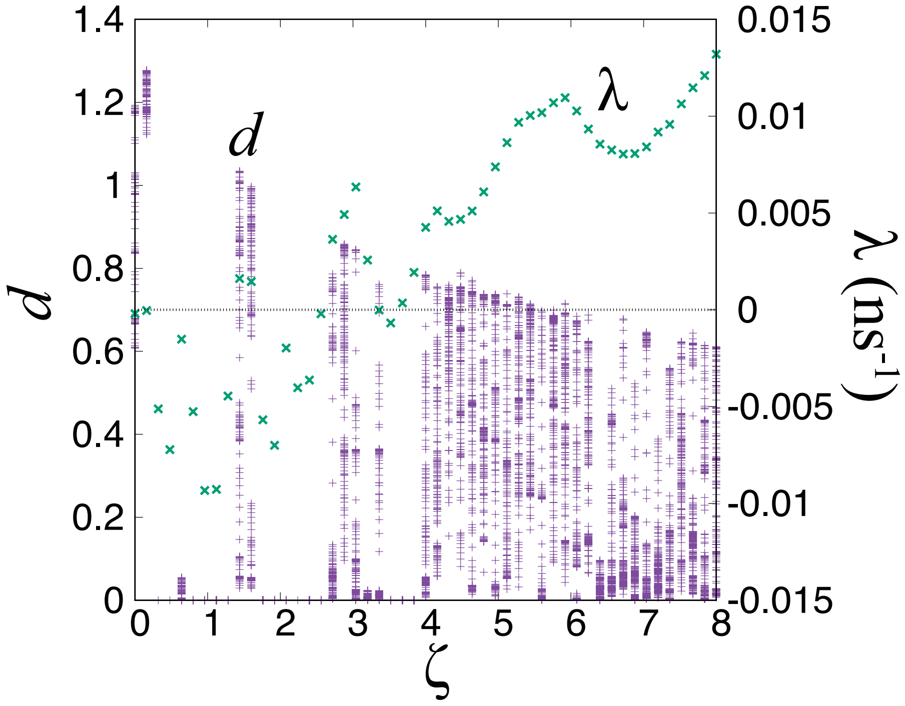

Figure S 4. The bifurcation diagram of the local maxima d of the distance $|\mathbf{X}_1 - \mathbf{X}_2|/R$ as a function of ζ. The Lyapunov exponent (λ) is also shown. The current, the input strength, and the pulse width are 5.0 mA, 16.0 Oe, and 30.0 ns.

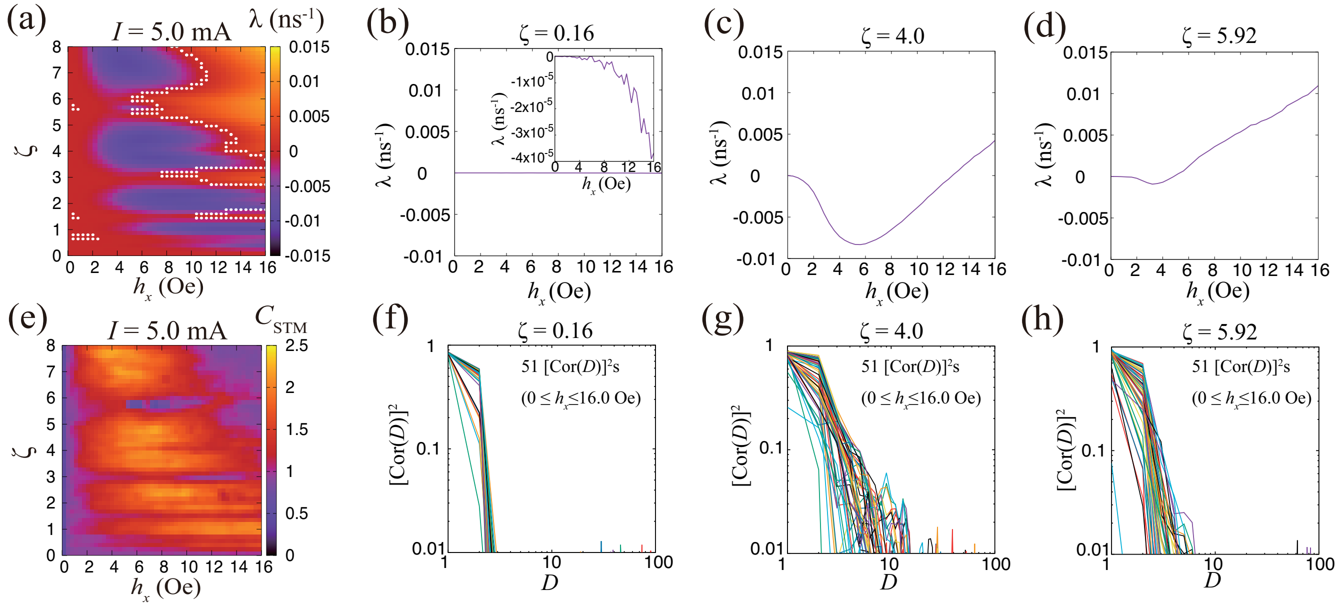

Figure S 5. (a) The Lyapunov exponent and (e) the short-term memory capacity for $I = 5.0$ mA and $t_p = 30.0$ ns [identical to that shown in Figs. S3(c) and S3(f)]. The exponents for $\zeta = 0.16$, 4.0, and 5.92 are shown in (b), (c), and (d), respectively. The dependencies of $[\mathrm{Cor}(D)]^2$ on the delay D for various h_x are shown in (f), (e), and (h), where ζ is 0.16, 4.0, and 5.92, respectively.

regions corresponding to chaos. Such window structures have been frequently observed in nonlinear dynamical systems[2,3].

Short-term memory capacity and the correlation coefficients near the edge of chaos

Remind that the short-term memory capacity is defined as

$$C_{\mathrm{STM}} = \sum_{D=1}^{D_{\max}} [\mathrm{Cor}(D)]^2, \tag{S.1}$$

where $\mathrm{Cor}(D)$ is the correlation coefficient for the delay D. Reference[4] performed numerical simulations of physical reservoir computing using an STO driven by an electric current and reported that $[\mathrm{Cor}(D)]^2$, called memory function, rapidly becomes zero with D increasing when the dynamical state of the STO is far away from chaos, while the memory function remains finite even for a large D when the STO is near the edge of chaos. Here, however, we show that this is not the present case.

Figure S5(a) shows the dependence of the Lyapunov exponent on h_x and ζ for $I = 5.0$ mA, which is identical to Fig. S3(c). From this, we plot the dependence of the exponent on h_x in (b) $\zeta = 0.16$, (c) 4.0, and (d) 5.92 of Fig. S5. These figures indicate the presence of the edge of chaos at which the sign of the exponent changes between negative and positive. Now, let us investigate the memory function near the edge of chaos. Figure S5(e) shows the short-term memory capacity, which is identical to Fig. S3(f). As mentioned above, the short-term memory capacity is the sum of the memory function, $[\mathrm{Cor}(D)]^2$. While the short-term memory capacity is evaluated for $D_{\max} = 20$ throughout this paper, let us investigate the dependence of $[\mathrm{Cor}(D)]^2$ on D for a large $D_{\max}$. Figures S5(f)-S5(h) show $[\mathrm{Cor}(D)]^2$ for $\zeta = 0.16$, 4.0, and 5.92, respectively, where there are 51 lines corresponding to h_x from 0 to 16 Oe with the step of 0.32 Oe. We notice that the dependence of $[\mathrm{Cor}(D)]^2$ on D is nearly the same for various h_x. This is in contrast with the result shown in Ref.[4] [Figs. 3(d) and 3(e) in the reference], where the memory function becomes sufficiently small for D around 3 when the STO is in an ordered state while it remains finite for $D \lesssim 30$ when the STO is close to the edge of chaos. The difference might relate to the fact that the short-term memory capacity in the present STO is not maximized near the edge of chaos.

References

1. Taniguchi, T., Ogihara, A., Utsumi, Y. & Tsunegi, S. Spintronic reservoir computing without driving current or magnetic field. *Sci. Rep.* **12**, 10627 (2022).

2. Strogatz, S. H. *Nonlinear Dynamics and Chaos: With Applications to Physics, Biology, Chemistry, and Engineering* (Westview Press, Boulder, 2001), first edn.

3. Yamaguchi, T. *et al.* Synchronization and chaos in a spin-torque oscillator with a perpendicularly magnetized free layer. *Phys. Rev. B* **100**, 224422 (2019).

4. Akashi, N. *et al.* Input-driven bifurcations and information processing capacity in spintronics reservoirs. *Phys. Rev. Res.* **2**, 043303 (2020).
